# Lupus Nephritis: An Overview of Recent Findings

**DOI:** 10.1155/2012/849684

**Published:** 2012-03-22

**Authors:** Alberto de Zubiria Salgado, Catalina Herrera-Diaz

**Affiliations:** ^1^Department of Internal Medicine and Clinical Immunology, The Samaritan University Hospital, Bogotá, Colombia; ^2^Center for Autoimmune Diseases Research (CREA), School of Medicine and Health Sciences, Universidad del Rosario, Carrera 24 No. 63C-69, Bogotá, Colombia

## Abstract

Lupus nephritis (LN) is one of the most serious complications of systemic lupus erythematosus (SLE) since it is the major predictor of poor prognosis. In susceptible individuals suffering of SLE, *in situ* formation and deposit of immune complexes (ICs) from apoptotic bodies occur in the kidneys as a result of an amplified epitope immunological response. IC glomerular deposits generate release of proinflammatory cytokines and cell adhesion molecules causing inflammation. This leads to monocytes and polymorphonuclear cells chemotaxis. Subsequent release of proteases generates endothelial injury and mesangial proliferation. Presence of ICs promotes adaptive immune response and causes dendritic cells to release type I interferon. This induces maturation and activation of infiltrating T cells, and amplification of Th2, Th1 and Th17 lymphocytes. Each of them, amplify B cells and activates macrophages to release more proinflammatory molecules, generating effector cells that cannot be modulated promoting kidney epithelial proliferation and fibrosis. Herein immunopathological findings of LN are reviewed.

## 1. Introduction

Systemic lupus erythematosus (SLE) is a systemic autoimmune disease in which diverse immunological events can lead to a similar clinical picture, characterized by a wide range of clinical manifestations and target organs (phenotypes) with unpredictable flares and remissions that eventually lead to permanent injury. Sociodemographic factors such as sex, race, and ethnicity play an important role in the incidence of the disease, frequency of its manifestations, and therapeutic response.

The overall prevalence and incidence of SLE ranges from 1.4 to 21.9% and from 7.4 to 159.4 cases per 100,000 people, respectively [1]. SLE can affect several organs and systems, including the joints, skin, brain, heart, lungs, blood vessels, and kidneys.

Lupus nephritis (LN) is one of the most serious SLE complications since it is the major predictor of poor prognosis. The incidence and prevalence of LN varies depending on the studied population. The LN cumulative incidence is higher in people of Asian (55%), African (51%), and Hispanic (43%) ancestry compared with Caucasians (14%) (1). Up to 25% of these patients still develop end-stage renal disease (ESRD) 10 years after onset of renal compromise [2]. In terms of outcome, the 5- and 10-year renal survival rates of LN in the 1990s ranged between 83–93% and 74–84%, respectively [2]. In addition, LN develops early in the course of SLE thus becoming a major predictor of poor prognosis [3]. However, in about 5% of the cases, LN may appear several years after the onset of SLE (i.e., delayed LN) [4]. The group with delayed LN is positively associated with Sjögren syndrome (SS), lung involvement, and antiphospholipid syndrome as compared with early LN (i.e., those SLE patients who develop LN during the first 5 years of the disease) [4].

**Figure 1:**
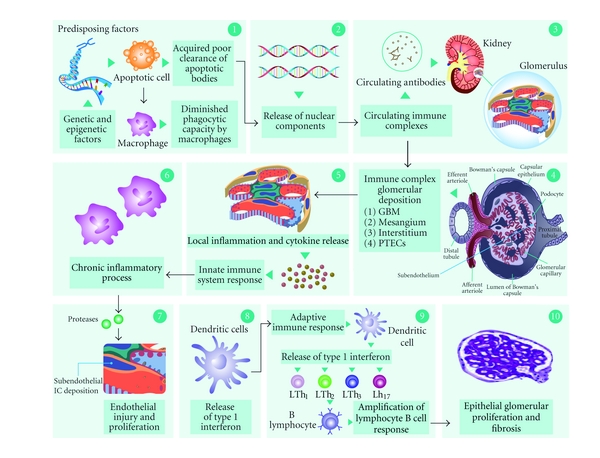
Lupus nephritis: an imbalance between cytokine homeostasis and immune complex deposition. In predisposing susceptible individuals who develop systemic lupus erythematosus (SLE), there is an acquired poor clearance of apoptotic bodies and a diminished phagocytic capacity by macrophages (1). Early formation of immune complexes (ICs) include antinucleosomes, anti-double-stranded DNA (anti-dsDNA), DNA extractable nuclear antigen antibodies (ENAS), antibodies against C1q complex of the complement system, free DNA, antiribonucleoproteins (anti-RNP), and histones as byproducts of inefficient phagocytosis of apoptotic bodies (2). Circulating ICs are deposited initially at the glomerular base membrane (GBM), mesangium, and interstitial tissue within the proximal tubular epithelial cells (PTECs) (3) and (4). The deposited ICs initiate the release of proinflammatory cytokines and chemokines such as monocyte chemoattractant protein 1 (MCP-1), interleukins 1 and 6 (IL-1, IL-6) and adhesion molecules (CAMs) thus establishing a chronic inflammatory process (5). The resulting overload of the mesangial phagocytic system (innate immune system) leads to deposits of subendothelial ICs becoming an easy target for monocyte migration and infiltration and generating endothelial injury and proliferation (6) and (7). In turn, the adaptive immune system is activated secondary to the presence of ICs and dendritic cells (DCs) (8), which subsequently trigger release of type 1 interferon and induce maturation and activation of infiltrating T cells. This activation leads to sequential amplification of T helper 2 lymphocytes (Th2), T helper 1 (Th1), and T helper 17 (Th17) (9). Each of these amplifies lymphocyte B cell response and further activates macrophages, generating a second general response, which increases recruitment of effector cells that can no longer be modulated by regulatory T cells and resulting in the end in epithelial glomerular proliferation and fibrosis (10).

LN has been looked upon as a classic example of immune complex-induced microvascular injury which results from circulating double-stranded DNA polynucleotide antigens/anti-DNA antibody complexes and other mechanisms including *in situ *reactivity for free antibodies with fixed antigens and the presence of sensitized T cells which are an important part of the picture [5]. Early deposits of immune complexes (ICs) include nucleosomes, DNA-extractable nuclear antigen antibodies (ENAS), and antibodies against C1q complex of the complement system as byproducts of inefficient phagocytosis of apoptotic bodies. This results in an autoimmune response through epitope expansion. These ICs have predominance over immunoglobulin G (IgG) 2 and 3. Deposits of ICs are initially located at the glomerular mesangium and interstitial tissue within the proximal tubular epithelial cells (PTECs) [5]. These deposited ICs initiate the release of proinflammatory cytokines and chemokines such as monocyte chemoattractant protein-1 (MCP-1) and cell adhesion molecules (CAMs) thus establishing a chronic inflammatory process. The resulting overload of the mesangial phagocytic system leads to deposits of subendothelial ICs becoming an easy target for monocyte migration and infiltration [5]. This migration and infiltration is due to a general response of the innate immune system that releases inflammatory proteases thus causing endothelial injury and proliferation. In turn, the innate immune system response promotes the activation of adaptive immune system secondary to the presence of ICs and dendritic cells (DCs), which subsequently trigger release of type 1 interferon and induce maturation and activation of infiltrating T cells. This activation leads to sequential amplification of T helper 2 lymphocytes, (Th2) T helper 1 (Th1), and T helper 17 (Th17). Each of these amplifies lymphocyte B cell response, and activates macrophages. This generates a second general response, which increases recruitment of effector cells that can no longer be modulated by regulatory T cells and, in the end, results in epithelial glomerular proliferation and fibrosis [5] (Figure 1).

## 2. Factors Influencing LN: Role of Ethnicity

So far, it has been difficult to predict the course of LN. Renal compromise in SLE has been markedly heterogeneous in terms of clinical presentation and course. One of the most important factors influencing LN is ethnicity. Prevalence in populations varies depending on ethnicity. In a recent case control study, Sisó et al. found an overall prevalence of 31% of LN in a large cohort of white Spanish biopsy-proven patients. One third of these patients developed end-stage renal disease (ESRD) [6]. Most studies have reported rates of up to 31% ESRD in Africans and 18% in Hispanics compared to 10% ESRD in Caucasians [7]. However, more than a decade ago, Molina et al. described African and Latin American patients with LN in a study with cohorts of 222 and 300 patients, respectively, which showed a higher prevalence of LN 46% for both populations [8].

 SLE patients from 9 different Latin American countries were evaluated in the GLADEL Multinational Latin American Prospective Inception Cohort of 1,214 Patients in 2004. Amongst the statistical significant results; Afro Latin Americans (ALA) mestizos had more severe disease than did whites, as evidenced by a higher frequency of renal disease, pericarditis, polyadenopathy, and discoid lesions in ALA. In addition, both ALA and mestizos had higher maximum disease activity indices than whites, but this was lost when controlled by country. However, damage scores tended to be lower in ALA than in both mestizos and whites, a surprising finding that might be explained by shorter disease duration or by the more recent incorporation of Brazilian and Cuban groups into the study. A peculiar observation was that of a significantly lower frequency of both xerophthalmia and sicca syndrome [8].

## 3. Murine Models

### 3.1. Spontaneous Murine Models

There has been a renewed interest in the use of animal models in the study of IC mediated LN, which has focused on immune and inflammatory mechanisms involved in the disease process. The majority of the murine models have been created to mimic LN [9]. This research has led to a better understanding of the disease by learning about the role of new cells and molecules that have been involved in the pathogenesis of LN. There are many known lupus murine models, which include spontaneous mice with inherited susceptibility, transgenic, and deletion knockout mouse models [9].

Specifically, three spontaneous lupus (inherited susceptibility) mouse models have been extensively studied: New Zealand Black (NZB), New Zealand White F1 mice (NZWF1), inbred strains of mice (BXSB), and mice homozygous for the apoptosis-defective Faslpr mutation (MRL-Faslpr). These models share some similarities with human SLE including the presence of antinuclear antibodies (ANAs), ICs, activation of T and B cells, and kidney disease. Nevertheless, there are sharp differences in the genetic origin and target organ involvement in murine models. The MRL mice are the result of a mutation of Fas with diminished apoptosis in lymphocytes, which generates hyper proliferation and secondary organomegaly [9, 10].

### 3.2. Transgenic Mice Models

Transgenic as well as deficient (knockout) models have clarified the function of many molecules as well as their potential role in autoimmunity. This, however, does not necessarily mean that these genes are relevant to human SLE. For instance, deletion of the Fc receptor in immunoglobulins (FcR) in NZB mice prevents injury despite the deposit of ICs [11, 12]. The above result is consistent with the fact that anti-DNA antibodies can modulate gene expression in mesangial cells through Fc-gamma-receptor- (Fc*γ*R-) dependent and independent pathways, which can induce proliferation, extracellular matrix synthesis, and production of proinflammatory cytokines [13, 14].

Transgenic models with deleted genes (knockout models) have altered tolerance to B cells or T cells. These gene deletions include Fc*γ*R, Bim, CD22, Lyn, (src-tyrosine kinase involved in B-cell activation) CD72, and co-stimulatory receptor (PD-1). In the MRL model, the removal of interactions of the programmed death 1/programmed death ligand 1 (PD-1/PD-L1) pathway provided a negative regulatory checkpoint in mediating tolerance and autoimmune disease. PD-L1 caused early death by autoimmune myocarditis and pneumonitis [15]. In addition, Lyn gene deletion in transgenic models affects the ability of B cell receptors (BCR) to edit. A T cell role has been demonstrated to be implicated in LN through the deletion of CD4+ T cells in transgenic models. The CD28 molecule, in turn, appears to be essential to initiate the activation of lymphocyte CD4 + T cells and also to induce costimulatory proteins (ICOS), which are more important in the activation of previously differentiated effector T cells. An induced deficiency of ICOS reduces autoantibody titers of IgG and *in situ *survival of T cells but does not affect the condition [16].

Natural inhibitors of the CD28/B7 pathway include the cytotoxic T-lymphocyte antigen 4 (CTLA-4) receptor in T cells and PD-1. Both of these recruit inhibitor protein tyrosine phosphatase (SHP-2). PD-1 chronically inhibits activated T cells and makes them respond in peripheral tissues but not in lymphoid organs. This is essential in maintaining T cell tolerance. The fine control between T regulator cells and PD-1 pathway may depend on the completion of an uncontrolled reactive autoimmune response [17]. The PD-1 pathway has the ability to simultaneously remove self-reactive T cells and promote the development of LT regulator cells. 

## 4. Genetic Susceptibility of SLE

Patients with SLE have defects in all branches of the immune system including innate immunity, antigen presentation, apoptosis, impaired tolerance in T and B cells, and defective release of regulatory cytokines and chemokines. SLE should be considered a failure of immune tolerance in one or more of the central or peripheral checkpoints with summation effects of multiple genes related to the immune response [18]. 

The tendency to self-reactivity is a natural phenomenon as it is estimated that 75% of recently formed B cells in the bone marrow in adults and 40% of the B cells located in germinal centers are autoreactive [19, 20]. In murine models, defects have been detected in both central and peripheral tolerance in B and T cells by introducing self-reactive receptors [21]. However, in humans a natural selection mechanism is currently believed to be the major one for reducing reactive immature B cells by as much as 75% in the bone marrow [22]. An altered edition of this mechanism has been reported in some patients with SLE. B cells that get through this defective mechanism will be subjected to control in the periphery by induced deletion, anergy, or apoptosis. Both biological processes require strong BCR signals that activate an inhibitory pathway via the CD22-tyrosine phosphatase SHP-1 thus avoiding clonal amplification through the inhibition of the interaction between B and Tfh cells [22]. 

For years now, human susceptibility to systemic autoimmunity has been related to several genes with polymorphisms or mutations that encode defective proteins involved in the immune system. HLA and non-HLA genes contribute to the polygenic susceptibility of the disease, and about 30 genes have been consistently replicated and confirmed to influence the predisposition of SLE. For instance, a genome-wide association study (GWAS) evaluating 317,501 single nucleotide polymorphisms (SNPs) in 720 women of European ancestry with SLE and 2,337 controls disclosed four loci associated with the disease harboring the following genes: *ITGAM*, *KIAA1542*, *PXK, *and the SNP rs10798269 in chromosome 1q25.1 [23]. In addition to the already established gene associations with SLE and other autoimmune diseases, *FCGR2A*, *PTPN22, *and *STAT4 *were confirmed. These results are only an example to show that several genes, some with known immune-related functions, predispose to SLE [23].

One of the most interesting genes associated with SLE is *PTPN22*. This gene encodes for the protein tyrosine phosphatase Lyp, in which a missense mutation that changes residue 1858 from cytosine to thymidine (1858C/T) is associated with multiple autoimmune disorders including SLE, rheumatoid arthritis (RA), and type 1 diabetes (T1D) [24, 25]. The protein, encoded under normal circumstances, is involved in B cell signaling. However, with the presence of autoantibodies associated with the 1858T variant, B cell signal transduction is impaired thus contributing to autoimmunity.

A polymorphic variant of *IRF5 *has been linked to SLE and high circulating levels of Type I interferon (IFN). The genetic alterations may lead to sustained overproduction of IFN *αβ* in human SLE, which will result in increased bioavailability and activation of immature DCs that control peripheral tolerance by deleting autoreactive lymphocytes [26, 27]. IFN mature DCs activate and expand autoreactive T cells thus helping autoreactive B cells to differentiate. In addition to its indirect effect through DCs, IFN also directly allows the expansion and survival of CD4+ and CD8+ T cells as well as the differentiation of B cells into plasma cells. The increased frequency of autoreactive B cells depends on a second set of genetic alterations that target B cell tolerance checkpoints. These early events create a first level of autoimmune injury, which is clinically silent but might generate apoptotic cells and nucleic acid-containing immune complexes. The capture of these apoptotic cells by myeloid DCs and nucleic acid-containing ICs by peripheral DCs and autoreactive B cells broadens the autoimmune reaction thereby leading to disease manifestations [26, 27].

Many of the genes associated with more severe forms of SLE such as *HLA* genes have also been associated with LN. Certain alleles in the HLA-DR2 and the HLA-DQ haplotypes seem to be particularly associated with LN in specific ethnic groups [28, 29]. In addition, in a cohort of 2,366 patients with SLE and 2,931 controls with common European ancestry, a variant at exon-3 (rs1143679 A) of Integrin-*α*-M (*ITGAM)* was strongly associated (*P* < 0.0003) with renal criteria in these patients. Among non-HLA genes associated with LN, *ITGAM *has been consistently reported to influence this SLE manifestation [30].

In African Americans, a strong risk factor has been associated with the presence of a monocyte receptor polymorphism in Fc*γ*RII-H131 that interacts with IgG2, which reduces the hepatosplenic clearance of circulating ICs [31]. In the context of the pathogenesis of LN, this may be important because it will facilitate glomerular IC deposit (Table 1).

## 5. Pathogenesis and Antibodies

### 5.1. Glomerular Immune Complex Deposit and Anti-dsDNA

An appropriate understanding of the current model of glomerular immune complex deposit is based on several experimental models of LN that use double-stranded anti-DNA antibodies (anti-dsDNA) with different affinities and physicochemical properties and correlate them in the eluate of patients with LN.

Apparently, renal involvement begins with the glomerular ICs deposit. These ICs are predominantly antibodies against single-stranded (ss) and double-stranded (ds) DNA as well as some polyreactive reagents that include anti-Sm, anti-RNP, anti-histones, anti-Ro/SS-A, anti-La/SS-B, and anti-C1q antibodies [5]. The formation of ICs seems to be predominantly *in situ*. However, although anti-dsDNA ICs are present in LN most of the time, it has not yet been proven that these types of ICs are enough to induce LN [69].

**Figure 2:**
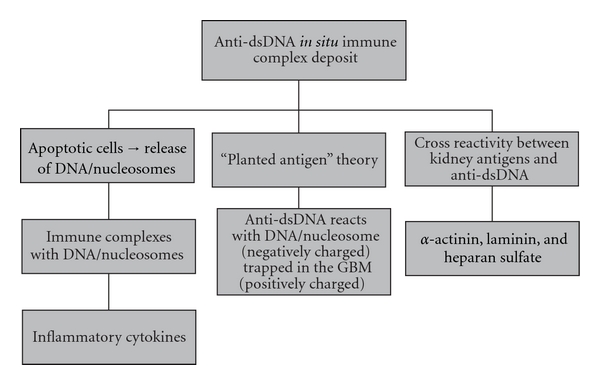
Proposed theories for anti-dsDNA *in situ *immune complex deposit [13]. GBM: glomerular base membrane.

Three mechanisms have been proposed to explain the ability of anti-dsDNA to settle in the kidney [13]. First, anti-dsDNA reactive antibodies can form ICs with DNA/nucleosome previously released from apoptotic cells. These ICs may be deposited in the kidney and initiate an inflammatory cascade. There is another postulated theory commonly known as the planted antigen theory. This theory proposes that anti-dsDNA reacts with DNA/ nucleosome trapped in the glomerular base membrane (GBM). In addition, the trapping of DNA/nucleosome has been associated with the negatively charged DNA and positively charged GBM. The third theory relates to the cross-reactivity between kidney antigens and anti-dsDNA. Nephritogenic anti-dsDNA antibodies have been shown to cross-react with alpha-actinin, laminin, and heparan sulfate (Figure 2).

The amount of deposited ICs, isotypes, and their affinity correlates with the severity of LN. The ICs located at the mesangium and subendothelium subsequently contribute to the recruitment of inflammatory cells. Although there is a predominant deposit of IgG and isotypes IgG2 and IgG3, there are also IgM and IgA deposits as well as C3, C4, and C1q molecules, which are part of the complement system [69].

The activation of the inflammatory cascade is achieved through Fc gamma receptors in macrophages, DCs, neutrophils, mesangial cells, and kidney cells [70]. It is also achieved by cross-reactivity with nephritogenic proteins expressed in renal parenchymal cells, PTEC, and mesangial cells thus generating the release of proinflammatory mediators and vascular adhesion molecules. Mesangial cells and PTEC are the most involved in releasing cytokines such as Interleukin-6 (IL-6), interleukin-1 (IL-1), tumoral necrosis factor (TNF), and chemokines such as MCP-1 [71]. It is worth highlighting that these nephritogenic compounds, as has already been mentioned, are related to the expression of laminin or collagen IV.

In addition, once ICs are deposited, they cannot be phagocytosed by mesangial cells and so will be deposited in the subendothelium. This leads to the first migration and posterior infiltration of monocytic effector cells and polymorphonuclear cells (PMNs). This cell recruitment is mainly mediated by the action of proinflammatory cytokines and by the complement system thus causing tissue damage [12]. This, in turn, increases the release of more proinflammatory cytokines (IL-1, IL-6, and TNF-*α*) and chemokines such as MCP-1, secreted cytokine (RANTES), TNF-related weak inducer of apoptosis (TWEAK), and activation of CAMs (ICAM-1, VCAM-1), all of which enhance amplification of the innate immune response. Moreover, the dysregulation in the synthesis of cytokines could be responsible for mesangial proliferation, crescent formation, and progressive glomerulosclerosis. The cytokines involved are IL-4, IFN-*γ*, transforming growth factor (TGF), platelet-derived growth factor (PDGF), and MCP-1 [12]. 

To support the idea that Fc*γ* receptors are directly involved in the activation of the inflammatory cascade, LN has been attenuated in the knockout models [72].

The adaptive immune response is simultaneously promoted by the presence of ICs, which causes a reaction within the DCs, and this induces the release of type I IFN. As a result of the subsequent maturation of the DCs, antigens are presented and infiltrating T cells undergo further activation. This leads to amplification of Th2 responses, Th1, Th3, Th17 and B cells and further activates a new wave of effector cells such as monocytes and PMNs.

Based on murine models and neonatal studies in class V NL (i.e., membranous), there is also an *in situ *glomerular deposit of ICs. In this case, the antibody recognizes the receptor of phospholipase 2 expressed in podocytes. However, the target antigen in class V LN has not yet been identified. The subepithelial ICs then trigger a cascade of events that generate podocyte injury with flattening and sloughing through the activation of the complement membrane attack complex (MAC). Ultimately, this disruption is responsible for proteinuria. In contrast to the endothelium and mesangium, podocytes do not proliferate in response to injury but produce thickening of the GBM due to increased synthesis of extracellular matrix proteins [73].

Aside from anti-dsDNA being directly involved in *in situ *IC formation, high-affinity anti-DNA plays a central role in some of the manifestations of SLE, especially LN. They are relatively specific and are good markers of activity in some patients. This has been confirmed in a large cohort of 1,000 patients reported by Cervera et al. [74]. Not all anti-dsDNA antibodies are related to LN. As mentioned before, this depends mainly on their specificity, affinity, isotype and idiotype, cross-reactivity with glycosaminoglicanos, and ability to interact with nucleosomes or DNA-linked collagen. The lack of IgM anti-dsDNA secretion is associated with apparently more severe LN [75]. However, the disease can develop in the absence of anti-dsDNA [13, 76].

### 5.2. Role of Complement in LN

Low total complement hemolytic activity and decreased C3 and C4 levels are detected in 75% of the patients with class III and 90% of those with class IV LN. The settling of IgG isotypes, IgA, IgM, C1q, C4, C3, and C5b-9 is called a *full house, which* is almost exclusive to LN. Complement degradation products such as C3d and C5b-9 can also be detected in urine thus providing circumstantial evidence of the role of the complement system in LN. However, C3 deficiency does not reduce the risk of LN and its true role is unknown [70]. Some studies suggest a predominant mechanism via Fc*γ* receptors [12, 77].

### 5.3. Antinucleosomes

Chromatin is the complex of histone-native DNA in eukaryotic cells. It is the packaging unit of DNA and controls the expression of genetic information by regulating access to transcription factors. There has been increasing evidence that nucleosomes are the main targets of the IC deposits [78].

Apparently, antinucleosome complexes adhere to heparan sulfate and have been detected in the human glomerulus. The main source of nucleosome release is from lymphocyte apoptotic bodies. It seems that they are generated at a very early stage even before DNA is released [79].

Free DNA has very few antigenic properties. It becomes more immunogenic as DNA-protein complexes show tridimensional epitopes of chromatin. Several histone fractions are shown to be able to bind glycosaminoglican proteins. It seems likely that the immune response begins with anti-nucleosome and anti-DNA antibodies and is the result of epitope amplification response. When these complexes are given to murines, they cause a lupus-like syndrome (SLE-like) [80]. Histone DNA complexes have a higher affinity for glycosaminoglycans *in vitro *and serve as a histone anchor for a larger deposit of DNA. Kalaaji et al. demonstrated antichromatin deposits in human and murine lupus LN by electron microscopy [81, 82]. This chromatin appeared to originate from glomerular apoptotic cells.

### 5.4. Anti-C1q

In 2004, Trouw et al. demonstrated in a mouse model that antibodies against C1q of the complement system (anti-C1q) play a pathogenic role in LN in the presence of ICs [83]. Anti-C1q could participate in glomerular injury by reducing the clearance of circulating ICs.

### 5.5. Alterations in Apoptosis

In healthy individuals, dead cells, mainly T and B cells as well as PMNs, are rapidly removed by macrophages in a noninflammatory context. In SLE patients, poor clearance of apoptotic bodies leads to the release of self-antigens that are subsequently submitted to T cell presentation by follicular DCs and B cells in secondary lymphoid organs thus challenging peripheral self-tolerance [84].

In 1998, mice exposed to syngeneic apoptotic thymocytes intravenously induced development of ANAs, anticardiolipin, and anti-ssDNA antibodies as well as deposits of ICs in the kidney [85]. Some of the autoantibodies generated react with nuclear products as a result of degradation by granzymes present in membrane vesicles of apoptotic cells [86]. This leads to the release of DNA-histone complexes, free DNA, small RNA, SS-A SS-B, and overexpression of phospholipid molecules in the membrane. The clearance of apoptotic cells is finely regulated through the activation of multiple receptors in phagocytic cells (scavenger receptor, phosphatidylserine receptors) that detect apoptotic cells [87]. A decreased ability on the part of macrophages to clear apoptotic bodies in a considerable number of patients has been previously described. This appears to be a defect since they have diminished phagocytic capacity at different stages of maturation [88, 89]. This defect alters the balance of peripheral tolerance and generates a first phase of autoimmune activation leading to a reaction of natural autoreactive B cells with subsequent epitope amplification [90].

A slight increase in apoptosis at the tubule-interstitial level that correlated with mononuclear infiltrates in 35 kidney biopsies of patients with LN was reported. In addition to these findings, the level of apoptosis of tubular cells had a positively significant statistical correlation with the activity index score for mononuclear cell infiltration but not with scores for other chronicity index components [91].

### 5.6. Dendritic Cells in LN

DCs are the most powerful antigen presenting cells (APCs) and are crucial in both innate and adaptive immune responses [92]. DCs are classified as type I or tolerogenic cells, which release interleukin 10 (IL-10), and type II or immunogenic cells, which release interleukin 12 (IL-12).

DCs are found in peripheral tissues where they capture antigens and then migrate to lymph nodes to exert their APC function on follicular helper T cells (fhT) by regulating the activation and differentiation of cell populations. They can also interact directly with B cells. DCs descend from two lines, myeloid and lymphoid. They differ in the expression of toll-like receptors (TLRs). Lymphoid DCs release cytokines such as IL-12 and IL-18. Myeloid DCs (mDCs) are the largest population and are differentiated from monocytes. Their synthesis rises in bacterial infections. Moreover, DCs can synthesize multiple cytokines and chemokines such as IL-1, IL-6, IL-8, IL-12, IL-18, granulocyte macrophage colony stimulating factor (GM-CSF), MCP-1, IL-10, and TGF. DCs are considered to be the largest producers of IL-18 and promote lymphocyte Th1 responses [92].

DCs are crucial for maintenance of immune tolerance. Circulating immature DCs capture antigens and migrate to lymph nodes, where they present self-peptides in the absence of costimulatory signals to T cells, which induce their anergy or deletion [93].

Human DCs instruct naïve CD4+ T cells to become IL-21-producing Fh T cells through the secretion of IL-12 [92]. IL-21 is a B cell growth factor required to induce differentiation and isotype switching and cooperates with IL-6 and the B lymphocyte stimulator (BlyS). In turn, IL-12 appears to induce both Th1 *γ* interferon production as well as IL-2 [92].

In SLE, DCs are activated by self-antigens through TLR 3, 7, 8, 9 or Fc*γ* receptors and are induced to release IFN-*α*, a crucial molecule in autoimmunity that also plays a key role in LN [94].

Several subtypes of DCs are detected in normal human kidneys, predominantly the myeloid cells. Only 25% of them are plasmacytoid DCs (BDCA2 +). In murine models of LN, DCs in GBM are increased in NZB mice. In proliferative forms of murine LN, a population increase has been shown in CD68+ myeloid/macrophage cells at the glomerular interstitium [85] as well as an increase in lymphoid DCs [95, 96]. Apparently, the extent of infiltration is higher in proliferative classes. Most of the DCs detected in LN are immature in contrast to SLE patients that show a marked reduction of mature DCs and lymphoid cells [95]. This could be the consequence of their migration to the kidneys and other tissues during the activity of the disease.

LN has been attributed to an imbalance between cytokine homeostasis and IC deposits. High synthesis of cytokines and chemokines by DCs may contribute to LN pathogenesis. Therefore, the increased migration of DCs, which has been recognized in the kidney may be due to the early release of IL-18, IL-1, and C-C chemokine receptor type 5 (CCR5) [97]. These, in turn, are central in regulating the secretion of more cytokines and chemokines and determining the prevalence of the response of Th1 and Th2 cells.

### 5.7. Role of T and B Cells in LN Pathogenesis

#### 5.7.1. T Lymphocytes

T cells are divided into effectors and regulators. The former includes CD4+ Th1, Th2, Th3, cytotoxic CD8+, and Th17. Regulator T cells include (FOXP3+ CD25+) T cells and natural killer T cells (NKs) [97]. Together, these cells participate in initiation, amplification, and regulation of the immune response in LN as well as migration, destruction, fibrosis, resolution, and exacerbations of the disease [97]. Therefore, they have become one of the targets for therapeutic intervention [97].

Central defects detected in SLE patients not only include the substitution or replacement of the T cell receptor (TCR)/CD3*δ* by TCR/Fc*γ* [97] but also display constitutive changes in the grouping of lipid vesicles that carry transcription factors with the consequence of early aberrant TCR signaling [98] and decreased threshold of activation. The molecular mechanisms of the above mentioned are not clear but appear to be both transcriptional and posttranscriptional modifications. In SLE, the entry of calcium activates calcineurin, which, in turn, activates the nuclear factor of activated T cells (NFAT) thus increasing the expression of CD40L and stimulating B cell activation and the synthesis of immunoglobulins [98].

In LN, activated CD4 and some CD8 T cells as well as activated macrophages and DCs infiltrate the renal interstitium, thus worsening renal function [99]. The restricted use of V*β* chains on recruited T cells suggests their oligoclonality and are potentially specific antigens or autoreactive [100]. Crispín et al. reported infiltrates of double negative (DN) T cells and Th17, which were presumably derived from a population of blood-producing IL-17 DN T cells [101]. T memory CD45RO+ expressing cells were also detected in the urine of patients with LN [102].

It is worth mentioning that DN T cells constitute a small population (less than 5%) in healthy subjects and are significantly increased in patients with SLE [101]. Having a mixed profile of Th1, Th2, and Th17, they synthesize IL-4, IL-17, IL-1, and IFN-*γ*. DN T cells are also found in patients with LN. However, why this cellular differentiation happens is not yet clear.

In LN, T cells are able to interact with epitopes like nucleosome histone complexes. T cells also help autoreactive nephritogenic B cells, modulate the differentiation of T cell subpopulations, recruit macrophages and NK cells, and induce renal cell damage through the release of cytokines or direct cytotoxicity [103]. T cells also activate proximal tubular cells and promote parenchymal fibrosis [103].

CD40 and CD40L interaction between B and T cells induces clonal expansion, which makes differentiation into plasma cells (isotype switching) possible. Interestingly, the use of CD40L monoclonal antibodies (mAb) in NZB mice has been shown to delay the onset of the disease, reduce the number of B cells, suppress the isotype switching, and decrease the titles of anti-DNA antibodies [104].

At first, clarifying the role of Th1 and Th2 cells in murine LN was given great importance but their biological or genetic modulation showed some inconsistent results [105]. Both populations appear to contribute to a greater or lesser extent since giving both IL10 and IFN-*γ* to the NZ and WNZ (BWF1) hybrid mice accelerated nephritis, and antagonism of the two delays the disease as the antagonism of IL-4 or IFN-*γ* does in MRL mice [105]. However, there is evidence of a mayor predominance of the role played by Th1 cells in the pathogenesis of LN since the lymphocytic infiltration is abolished in the knockout model [106, 107]. The IFN-*γ*, in contrast, facilitates the interaction between T cells and parenchymal cells, especially PTEC, and increases the expression of HLA class II and accessory molecules [16].

Some of these contradictory results seem to depend on the confusing effect generated by the action of Th17 cell products, which are, in turn, promoted by the action of IL-6, IL-23, and TGF-*β* [108].

When IL-18 is administered to mice, LN is accelerated and the accumulation of DN T cells is fostered. As a result, IFN-*γ* is synthesized and DN T cells are differentiated into CD4+ cells and CD8+ cells. IL-18 antagonism reduces lymphoproliferation, production of IFN-*γ*, and progression of LN thus also implying a role for Th1 cells [109]. In addition, serum levels of IL-18 nearly double in patients with LN [110].

Microarray analysis suggests that production of nephritogenic autoantibodies in murine models depends on Th1 cells [111]. IFN-*γ* promotes the switch from IgG2 to IgG3, which is typical of LN unlike a predominance of IgG1 in the skin in SLE [112]. Therefore, IFN-*γ* seems to be crucial in modulating the activity of LN in murine models and promoting the synthesis of IgG2 in MRL-Faslpr and NZB mice [111].

Likewise, the expression of genes in infiltrating kidney T cells strongly suggests the presence of dominant Th1 though there is also some expression of Th2 GATA-3 cells (transcription factor) [113]. Chan et al. reported T bet (Th1 transcription factor) overexpression, IFN-*γ*, IL-2, IL-12, IL-18, MCP-1, and IL-10, which had a significant correlation with the histological activity index of LN [113]. Therefore, measurement of pro-Th1 in urine can be a promising biomarker for LN activity. This pro-Th1 response appears to be associated with proliferative LN class III and IV [114] and induces the switch towards Th1 response. This apparently worsens the disease and correlates with the histological activity index [115, 116]. In contrast, Th2 response appears to be predominant in Type V membranous LN models [117].

Nevertheless, the role of T cells in humans in the course of LN is less clear, and it cannot be resolved on the basis of murine models of gene deletion or costimulation. Other authors have confirmed the proliferative LN Th1 dominance in humans [114, 115]. However, in pediatric LN, a balance between Th1/Th2 on the basis of IgG subclasses has been detected [118, 119]. In proliferative LN, there is overexpression of TNF-related apoptosis-inducing ligand (TRAIL) in the glomerular tubules of patients [120]. These findings may play a protective role by enhancing PTEC survival while also exerting a proinflammatory effect that may contribute to local inflammation and injury by inducing expression of ICAM-1 and IL-8, which may also be caused by TNF-*α* and IFN-*γ* [120].

#### 5.7.2. B Lymphocytes

B cells are also abnormal and hyperactive in SLE. The uncontrolled activation of B cells may be the result of aberrant editing, increased signaling, an increase in co-stimulatory receptors B7 and CD40, increased subpopulations of plasmablastic DCs and plasma cells in the blood, and alterations of cytokines (BAFF, IFN-*α*, IL-6, and IL-21). The B-cell-activating factor (BAFF) rescues autoreactive B cells from deletion and induces isotype switching to IgG [121–124].

There has recently been a resurgence of interest in the role played by effector B cells not only through the synthesis of autoantibodies but also as regulators. To support this, some autoimmune models that were thought to be primarily mediated by T cells have shown potential roles for B cells through gene deletion or administration of CD20 monoclonal antibody in mice (independent autoantibody effects) [125].

B cells can also modulate some cellular responses by direct interaction with memory T cells and regulation of DC development. Indirectly, B cells are involved in cytokine synthesis: IL-10, IL-4, IL-6, IFN-*γ*, IL-2, IL-12, IL-23, IL-27, and BAFF. Under inflammatory conditions, they can function as B regulatory cells by releasing IL-10 and TGF-*β* through TLR stimulation. The increase in plasmablastic cells and B lymphopenia has been correlated with SLE clinical activity [125].

In humans, B cells seem to have some degree of organization rather than being random. Formation of ectopic germinal centers with organized follicles and DCs that correlate with the severity of tubule interstitial disease and deposit of ICs has been demonstrated as well [126]. However, to some authors, there is a predominance of the APC phenotype rather than synthesis of immunoglobulins as well as an increased expression of receptors for chemokine-type CXCR5 BCA-1 [127].

One of the most recent findings regarding B cells relates to circulating levels of BAFF (BLyS) in SLE, RA, and Sjögren's syndrome (SS). BAFF seems to contribute to B cell survival in germinal centers in a high percentage of patients and in NZB and MRL models. In these, it correlates with the amount of proteinuria and anti-DNA levels. BAFF acts synergistically with IL-6 and IL-21 to foster survival and differentiation of B cells in humans. It is synthesized by monocytes, neutrophils, DCs, and T cells [128, 129].

Interestingly, in transgenic murine models with BAFF over expression, there is an induced lupus-like syndrome with LN even in the absence of T cells [130, 131]. For some reason, it appears to mostly favor the maturation of autoreactive clones. Both plasmablastic and plasma cells express the receptor that is involved in the homeostasis of peripheral B cells [132].

Results from a GWAS pointed to B cell having an important role in the development of SLE through signaling and the involvement of TLR 7 and TLR 9 [67]. In SLE, the role of T cell regulators CD4+CD25 + Fox P3 has been demonstrated to suppress activity of B cells *in vitro *and *in vivo *[133].

#### 5.7.3. Th17 Lymphocytes (LTh17)

LTh17 are a subpopulation of CD4+ T cells and a subtype of high-producing IL-17 derived from Th1 cells [134]. LTh17 do not lend themselves to regulation by T regulator cells (Tregs) [135]. LTh17 do not lend themselves to regulation by T regulator cells (Tregs) [136]. The differentiation of naive cells into this proinflammatory Th17 subtype apparently occurs inversely to the development of Treg cells. Although both populations are induced by TGF-*β*, Th17 require the presence of inflammatory signals like IL-6, IL-21, and IL-23 as well in order to favor their differentiation and inhibit the Treg cells. In humans, it seems Th17 cells also synthesize IFN-*γ*.

When Th17 cells produce IL-17 in response to TGF-*β*, they activate kappa beta nuclear factor (*κβ*NF). Consequently, a MAP kinase cascade is generated and activates the ROR transcription factor [135]. This exerts a powerful proinflammatory effect and fosters increased recruitment of macrophages and neutrophils thus inducing the production of IL-8 and MCP-1. ROR transcription factor also induces CAM expression on T cells and the production of IL-6 and GM-CSF. This is how a second phase of inflammation is generated and becomes self-perpetuated. Th17 subpopulations do not appear to be antigen specific [135].

Zhao et al. evaluated IL-17 serum levels in fifty-seven patients with confirmed SLE and 30 healthy volunteers [137]. They found significantly elevated levels in patients with SLE. However, there was no positive association with activity of the disease measured by Systemic Lupus Erythematosus Disease Activity Index (SLEDAI), which indicates that there is still no concluding data regarding the role of Th17 and SLE and, therefore, LN [137, 138].

#### 5.7.4. T Regulatory Cells

The concept that Treg play an important role in maintenance of autoimmune response is well accepted. A decreased number and function of Treg cells implicated in murine lupus have been shown. However, human studies, many of which appear to be the result of clinical activity or immunosuppressive therapy [139, 140], are inconclusive [141, 142].

#### 5.7.5. T Cells and Adhesion Molecules

Cell adhesion molecules seem central and CD44 is greatly increased in patients with SLE [143]. Through alternative splicing and posttranslational mechanisms, this gene has several isoforms such as CD44v3 and CD44v6 that are high in SLE patients and correlate with the disease activity and the presence of LN. Infiltrating T cells express these isoforms. Patients with active NL have high urinary concentrations of VCAM-1. Their expression is regulated by IL-1 and TNF [144].

### 5.8. Effector Cells and Molecules

In proliferative LN, there is predominance of mononuclear infiltrates and, to a lesser extent, of neutrophils and platelets. Mononuclear activation depends on chemokines, complement system activation, and ICs and cause cytotoxicity in the target organ. When there is cytotoxicity, mononuclear cells become effector cells [97].

Also involved in LN immunopathogenesis, proteases that have been detected in urine of LN patients are presumably involved in the degradation of extracellular matrix proteins of the GBM and mesangium (serine proteases, elastases, cathepsin G, and collagenases) thus generating tissue necrosis [97].

In LN, many PMNs are located close to the crescents and their proteases and oxygen radicals and derivatives of nitrogen may contribute to tissue damage and necrosis. One of the proteases, collagenase B-associated lipocalin has recently been reported as a good biomarker of active LN [97].

In addition to releasing free radicals, nitric oxide, and proteases, macrophages also release proinflammatory cytokines such as IL-1, TNF-*α*, and IFN-*α*, PDGF, TGF-*β*, complement components, coagulation factors, and chemokines [97].

There is a prominent recruitment of type II activated macrophages mainly in the tubules, interstitium, and glomeruli both in murine models and in humans [145]. Their activation may also be enhanced during Th1 responses. The release of growth factors by macrophages may contribute to mesangial proliferation (PDGF and TGF *β*) and sclerosis in LN and MRL [146, 147].

### 5.9. Role of Intrinsic Renal Cells in LN

The main intrinsic kidney cells include mesangial, endothelial, and epithelial cells. Apparently, they are not innocent bystanders but may be signal amplifiers. This has been observed in murine models and appears to contribute on three levels: proinflammatory mediator release, fibrogenesis, and possibly APC.

Kidney mesenquimal cells (mesangial, tubular epithelial, and endothelial cells) synthesize and release significant amounts of MCP-1. They may also overexpress *α* actinin in the presence of IFN *γ* and IL-1. All of the above has been previously shown in murine models [148].

### 5.10. Cytokines, Chemokines, and LN

Although the picture is still unclear in terms of proinflammatory molecules, an upregulation of cytokines such as TNF, IL-1, IL-6, IL-18, IFN-*γ*, and IL-4, induce Th1 and Th2, cells, respectively. In contrast, a downregulation of TGF-*β* in an inflammatory context has been proven [149].

### 5.11. Interferon *α*

IFN type I or *α* is produced by all cell types but particularly by DCs in response to viral stimuli and in the presence of ICs [150]. Both pathways act by stimulating the TLRs types 3, 7, 8, 9 and thus induce maturation in DCs through the increase in the expression of costimulatory molecules such as ICAM-1, CD86, HLA class I and class II molecules. IFN-*α* activates hundreds of genes including viral transcripts (OAX, MX1), the IFN regulatory factor *(IRF) 5 *and *IRF7*, BLyS, chemokines (MCP-1 and IP-10) and enhances Th1 responses by inducing the synthesis of IFN-*γ* and expression of CXCR3 cells. IFN-*α* is a potent inducer of BlyS [151].

In SLE patients, a microarray analysis of thousands of genes has shown an over expression of IFN-*α*-inducible genes in about 40% of patients [152]. Specifically, three inducible genes showed a high IFN score and had a significantly higher prevalence of renal disease, increased SLE activity and presence of antibodies specific to Ro, U1 RNP, Sm, and ds-DNA but not phospholipids [94]. Steroid pulses dramatically decrease IFN-*α* expression by inducing significant depletion of pDC but their action is of short duration (one week) [153].

IFN-*α* seems to be a potent inducer of mesangial proliferation in the kidney, and several studies report that IL-6 is a critical mediator of the production of human nephritogenic antibodies [154].

### 5.12. IFN-*γ* in LN

It has been suggested that, in addition to inducing *in situ *synthesis of autoantibodies, IFN-*γ* increases the expression of CD40 molecules as well as that of MCP-1, ICAM-1, and VCAM-1. This seems fundamental in the pathogenesis of exacerbations of LN.

Recent recognition of the role of pro-Th1 cytokines such as IL-18, IL-12, and IL-27 makes this molecule central to the regulation of these responses [155]. According to multiple studies, higher circulating levels of IL-18 are found in patients with SLE and LN and may be crucial in the development of Th1 responses [156].

### 5.13. Interleukin 10

In patients with SLE, high levels of IL-10 are from three to twelve times higher than in controls, but there seem to be little correlation with the disease activity [157].

### 5.14. Transforming Growth Factor *β*

TGF-*β* ligands signal and activate intracellular effectors thus regulating transcription. TGF-*β* is a cytokine involved in both normal renal function and the development of glomerulosclerosis. It is produced by NKs, lymphocytes, monocytes, macrophages, and renal mesangial cells. It also has a stimulatory effect on T cells and a downregulatory effect on antibody production. In human SLE, several studies demonstrated the nephrotoxic effects of TGF-*β* within kidney cells. There is a strong relationship between expression of TGF-*β* and podocyte depletion and apoptosis. TGF-*β* also increases epithelial to mesenchymal cell transdifferentiation, induces peritubular capillary loss, and causes glomerular endothelial cell apoptosis. In contrast, the cytoprotective effects are mediated by the hepatocyte growth factor (HGF). Therefore, studies found that the balance between TGF-*β* and HGF seems to be an important prognostic factor in LN TGF-*β* and HGF [158, 159].

### 5.15. Interleukin-4

The IL-4 role in the synthesis of autoantibodies in murine models and in humans is controversial in LN [160, 161]. It also seems to promote the depositing of collagen type III in human mesangial cells and could contribute towards renal failure progression [162]. CD4+ cells, which produce IL-4 in patients with Class III and Class IV LN, were demonstrated by immunohistochemistry [163].

### 5.16. Chemokines

*Ex vivo *monocytes have shown increased synthesis of chemokines that correlate with SLE activity: interferon-gamma-induced protein 10 (IP-10), RANTES, monokine induced by IFN-gamma (MIG), MCP-1, and IL-18. They also seem to correlate with LN activity as does IL-8 [164].

Of the above molecules, the best studied is MCP-1 and more recently TWEAK. Chemokines not only play a preponderant role in inducing and regulating the selective chemoattraction but also participate in the regulation of cellular activation and exert angiogenic, fibrogenic, and hematopoietic effects.

There is increasing evidence that MCP-1 plays a role in the progression of renal failure based on different mouse models and in various proliferative LN [148, 165]. In murine knockout MRL, there is marked prolongation of survival and no monocytic or lymphocytic infiltrates. They seem to be the initiator of locally produced early tubule interstitial damage. As a result, MCP-1 is synthesized mainly by mesangial cells but also endothelial, mononuclear, and tubular epithelial cells and excreted in the urine. Therefore, MCP-1 in the urine of patients is a promising biomarker of LN activity [166]. In addition to its chemoattractant and releasing properties on mononuclear cells, MCP-1 appears to play a role *in situ *in inducing renal tubular and mesangial cells to synthesize proinflammatory cytokines such as IL-6, adhesion molecules, for instance, ICAM-1, and promote transcription of NF *κβ* by PKC [165]. In addition to that, MCP-1 promotes mesangial and endocapillary proliferation.

Chemokine receptors on T cells participate in regulating their trafficking. Native T cells express primarily CCR4, which interacts with chemokine C-X-C motif ligand 12 (CXCL12). Its pathogenic role has gained much attention recently. There is over expression in kidneys from NZBW, MRL, BXSB models as well as in LN in humans [167]. The use of murine C-C chemokine receptor type 4 (CCR4) antagonists has reduced the number of phenotypes and the use of C-C chemokine receptor type 3 (CCR3) antagonists reduced the infiltration of LTh1 and LTh17 and, therefore, the production of IFN-*γ* [168].

High expression of CXCR3 in 60% of infiltrating cells in biopsy material at the tubule-interstitial level has been recently reported in human type IV LN [169]. The CXCR3 receptor is a great candidate to explain the influx of LTh1 cells in LN. There are three CXCR3 ligands (CXCL9, CXXL10, CXCL11) with CXCL10 (IP-10) being the most potent inducer of IFN-*γ* synthesis. It has been proven to be a major chemokine expressed early on or preceding inflammation in murine LN [170, 171]. It is produced by endothelial cells, fibroblasts and monocytes stimulated with IFN-*γ*. In human LN, it appears to identify class IV nephritis [134]. In SLE patients, levels of IP-10 are very high and correlate significantly with the histological activity index. The expression of CCR5 may also play a role in Th1 chemotaxis [40]. CXCL16 is among other chemokines involved [172].

### 5.17. TNF Superfamily Cytokine (TWEAK)

This is widely expressed in human kidneys, specifically in mesangial cells, podocytes, and tubular cells. It is a proximal inducer of chemokine release. It also induces proliferation of mesangial cells and podocytes [173]. TWEAK has been recently studied as a biomarker for LN, and results have shown promising and significant results [174].

In summary, it is, therefore, possible that the deposit of ICs triggers the release by mesangial and tubule interstitial cells of MCP-1, TWEAK, and proinflammatory cytokines, which will contribute to the chemotaxis and after that, activation of monocytes and macrophages. This activation, in turn, releases chemokines such as CXCL10, which favors type LTh1 chemotaxis. LTh1 chemotaxis releases IFN-*γ*, which amplifies a further increased production of proinflammatory cytokines by monocytes and promotes IgG2 and IgG3 subclass synthesis. These immunoglobulins are responsible for generating anti-DNA antibodies, which induce glomerular cell proliferation.

## 6. Pathology

Classification of LN is critical to the issue of patient care and helps the physician make therapeutic decisions, follow up on the patient, and compare outcome results. In May 2002, a consensus conference of nephrologists, pathologists, and rheumatologists was held in order to redefine the different LN classes, and the meaning of the pathology terminology in order to standardize the way biopsies are interpreted and reported by different centers worldwide [175] (Table 2). The detailed pathological characteristics and their descriptions are beyond the scope of this paper. Readers are invited to consult pertinent and recent references about this topic [176].

### 6.1. Tubulointerstitial Disease

Active glomerular lesions have abundant interstitial inflammatory infiltrates of CD4+ cells and some CD8+ T cells, abundant monocytes, and plasma cells [99], which correlate with glomerular filtration rate and creatinine levels [177]. Others have found correlation between interstitial IC and serological activity [162]. Typically, the tubular damage, fibrosis, and atrophy are related linearly with renal function and are less responsive to treatment. These lesions often coexist with Classes III and IV.

## 7. Recent Advances in LN Therapy

LN impacts the clinical outcome of SLE both directly, in the form of target organ damage, and indirectly, through adverse effects of therapy [178]. On the other hand, the histological patterns of LN provide the basis for therapeutic guidelines and decisions to prevent target organ damage, as they are predictive. Despite improvements in survival rates and ESRD as mentioned before, LN is a marker of a bad prognosis [179]. Recent advances in therapy include a number of randomized controlled trials (RCTs), in which the goal has been to achieve clinical efficacy by inducing a remission of LN while at the same time minimize severe side-effects of treatment. The concept of two phases of therapy, an induction phase and a maintenance phase, is still widely accepted [180].

Patients with Class II LN and I do not require directed immunosuppressive treatment, and usually maintenance of adequate blood pressure control and blockade of the rennin angiotensin aldosterone system is the cornerstone of treatment. Patients with LN treated with ACE inhibitors have a better rate of renal involvement-free survival at 10 years (88.1%) as compared to patients with ACE inhibitors with a rate of renal involvement-free survival at 10 years of 75.4% (*P* < 0.01) [181].

### 7.1. Induction Therapy for Proliferative LN

Most patients with active proliferative LN are initially treated with a pulse of an intravenous steroid followed by a high-dose oral steroid, or by this method in conjunction with other immunosuppressive agents. These include cyclophosphamide, mycophenolate mofetil, and azathioprine.

#### 7.1.1. Cyclophosphamide (CY)

RCTs held at the National Institutes of Health (NIH) have provided strong evidence for the efficacy of IVCY in the treatment of proliferative LN. An IVCY pulse (0.5–1 g/m^2^) each month for six consecutive months followed by a follow-up pulse of low-dose corticosteroid every third month has been shown to be effective and prevent relapses better than a shorter regimen limited to the six monthly doses of IVCY alone [182].

The Euro-Lupus Nephritis Trial (ELNT) was a multicenter European RCT in which 90 patients with proliferative LN were randomized to either high-dose IVCY (0.5–1 g/m^2^) in 6 monthly pulses followed by two additional quarterly doses or to low-dose IVCY (500 mg) every 2 weeks to a total of 6 doses followed by azathioprine (AZA) maintenance therapy (2 mg/kg daily). After a median follow-up period of 41 months, there was no difference between the two groups in the rate of achievement of renal remission or in the rate of renal relapse [183]. The results of the ELNT of the follow-up period (73 months) showed similar results [184].

#### 7.1.2. Mycophenolate Mofetil (MMF)

The active metabolite of MMF suppresses B- and T-cell proliferation due to the absence of the salvage pathway necessary for DNA synthesis. That is the reason why results of several recent controlled trials have led to MMF being recommended as one of the first-choice regimens for inducing a remission in active proliferative LN. Chan et al. [185] randomized 42 patients with diffuse proliferative LN to either 12 months of oral MMF (2 g daily for 6 months followed by 1 g daily for 6 months) or to 6 months of oral CYC (2.5 mg/kg daily) followed by oral AZA (1.5 mg/kg/day) for 6 months, and both groups also received oral prednisolone (0.8 mg/kg). After a median follow-up period of 12 months, there were no significant differences between the remission rates (81 versus 76%), partial remission rates (14 versus 14%), or relapse rates (15 versus 11%) for both treatments; however, infections were less common in the MMF group. 

The Aspreva Lupus Management Study (ALMS) reported by Appel et al. [186] was one of the largest RCTs of a treatment of proliferative LN ever reported involving 370 patients with WHO class III, IV, or V LN randomized to 24 weeks of treatment with either MMF (3 g daily) or IVCY (0.5–1 g/m^2^). Both groups were also treated with prednisolone that started at 60 mg daily and was tapered. After 6 months of therapy, there was no significant difference between the two groups in the combined complete remission plus partial remission rates. Moreover, there was no difference in mortality between the two groups, and a total of 14 of the 370 patients died [186]. 

Overall, RCTs have shown no real difference in induction therapy for LN between CY and MMF in terms of complete and partial remission rates. However, infection rates as adverse events of immunosuppressants are lower with the use of MMF leaving to the physician's choice whether to start MMF or CY as induction therapy in order to achieve remission and prevent progression of renal disease.

#### 7.1.3. Tacrolimus

Recent findings regarding treatment in LN involve tacrolimus, which is a macrolide calcineurin inhibitor that potently suppresses human T-cell proliferation by inhibiting the intranuclear translocation of cytoplasmic nuclear factors in activated T cells by binding to tacrolimus-binding proteins and inhibiting calcineurin. Miyasaka et al. [187] reported a RCT that was undertaken to evaluate the efficacy and safety of tacrolimus in patients with persistent LN patients treated with a glucocorticoid. This RCT showed significant decrease in LN disease activity index (LNDAI) with tacrolimus when compared to placebo. A case-control study conducted by Szeto et al. [188] compared tacrolimus with standard protocols of oral CYC or AZA for the treatment of class V LN. Complete remission rate and partial remission rate were 38.9% and 44%, respectively, in the tacrolimus group, and 36.8% and 57.9%, respectively. It is important to remark that no significant adverse effects occurred in the tacrolimus group.

Five open-label prospective studies of the treatment of LN have been conducted [189–193], with preliminary evidence regarding the use of tacrolimus as induction-phase therapy. However, there is a need to conduct RCT with more proliferative LN patients in order to evaluate results and establish tacrolimus as on-label frequent use for the treatment of LN.

### 7.2. Maintenance Therapy for Proliferative LN

Maintenance therapy for proliferative LN focuses on maintaining renal remission previously achieved in the induction therapy. By avoiding flares or relapses, progression of renal disease can be achieved and, therefore, ESRD. The MAINTAIN Nephritis Trial [194], conducted on 105 patients with proliferative LN, was randomized to maintenance-phase therapy with AZA (target dose 22 mg/kg daily) or MMF (target dose 2 g daily). The MAINTAIN Nephritis Trial was predominantly Caucasian, and the results may not be applicable on populations of different ethnicities. Some meta-analyses have unequivocally favored the additional benefit of treating with immunosuppressive agents during the maintenance phase of LN therapy [195–197]. The selection and dosage in order to reduce long-term toxicities especially in childbearing age women must be done along with the patient. In addition, it is important to highlight the role of corticosteroids as a major component of treatment in the maintenance phase of LN therapy, despite the side effects of long-term steroid use.

### 7.3. New Agents for the Treatment of Lupus Nephritis

#### 7.3.1. Rituximab

This biological agent is a chimeric half murine-half human monoclonal antibody directed against the B cell marker CD20. Label indications of this biologic agent include RA and more recent SLE. Catapano et al. used Rituximab to treat 31 patients with relapsing or refractory SLE, 2 of whom developed relapsing/refractory LN during treatment with Rituximab (375 mg/m^2^/week for 4 weeks in one patient and 1000 mg × 2 doses in the other) [198]. After a 30-month follow-up period, peripheral B cells had been depleted in 97% of the patients, and a remission had been achieved in 87% of the patients (complete in 17 and partial in 10) [198]. A renal remission occurred in 10 of the 11 patients with active LN. Clinical improvement was manifested by reductions in disease activity, proteinuria, and daily prednisolone dose. A relapse occurred in 67% of the patients treated after a median interval of 11 months. In 50% of the patients who experienced a relapse, the relapse was associated with the return ossf circulating B cells. A second course of treatment with rituximab was effective. A recent systematic review, which covered the period from 2002 to 2007, demonstrated that 171 (91%) of the 188 patients with SLE treated with rituximab for severe, refractory disease had a significant improvement in at least one lupus manifestation, and 94 (91%) of the 103 patients with LN exhibited a therapeutic response [199].

There is more need for RCTs using biological agents such as rituximab and other agents that are in course of study like Belimumab and Abatacept. One may infer due to the important role of B cells and T cells in LN pathogenesis that directed target therapy against them could bring new insights for effective treatment in LN.

## 8. Conclusion

LN is considered to be the major complication or outcome in SLE. Its incidence varies widely between populations. Over the years, a better understanding of immunopathogenesis and natural history has developed, which ultimately results in effective therapeutic decisions for the benefit of the patient and prevent end-stage renal disease. In addition, this appropriate comprehension of NL gives hope to future therapy aimed directly towards specific cells, autoantibodies, cytokines, and chemokines in order to regulate inflammation and tissue injury.

LN results from a complex interaction between autoantibodies in association with anti-dsDNA, nucleosomes and histones that end up forming kidney ICs and permanently activated inflammatory cells that stimulate and induce proliferation in local cells, which, in turn, stimulate complement, cytokines and chemokines.

So far, therapy for LN has shown to be partially effective in terms of renal remission. Directed target therapy against B and T cells could bring new insights for real effective treatment in LN and thus achieving a better outcome in patients.

## Figures and Tables

**Table 1:** Adapted from [32] and [33]. Susceptibility genes in SLE associated with LN.

Chromosome	Gene	SNPs	Population	OR^*¹*^ with LN	References
6p21	HLA region	DRB1*0301 and several other Alleles.	European, Several Asian, African American, mixed European-Amerindian, and Latin American.	2.4	[29, 34–37]

7q32	*IRF5*	5bp promoter indel, rs2004640, rs2070197, 10954213 rs10954213 rs 729302	European, several Asian, mixed European-Amerindian, African American, Latin american.	1.6	[23, 38–42]

2q32	*STAT4*	rs7574865, rs3821236, rs7582694	European, mixed European-Amerindian, several Asian, African-American	1.5	[23, 38, 40, 43, 44]

6q23	*TNFAIP3*	rs5029939 rs223096 rs223096	European, Asian, African American	2.0	[23, 38, 40, 45–48]

16p11	*ITGAM*	rs9888739, rs1143679, rs4548893	European, mixed European-Amerindian, Asian, African American, Latin Americans	1.6	[30, 38–40, 46, 49, 50]

4q24	*BANK1*	rs10516487 rs1726654 rs3733197 rs1051647 rs10516483	European, European-Amerindian, Asian, Caucasian	1.2	[38, 46, 51–53]

1p13	*PTPN22*	rs2476601	European, Latin Americans	1.4	[23, 54]

8p23	*BLK*	rs13277113, rs2736340 rs2248932	European, several Asian	1.3	[23, 38–40, 46, 55, 56]

2q37	*PDCD* * (CD279)*	PD1.3A	European, European-Amerindian, Chinese, Latin Americans	1.2	[57]

1q25	*TNFSF4*	Risk haplotype; rs3850641	European, Asian	1.4	[23, 38, 40, 46, 52, 58]

18q22.3	*CD226*	rs763361 rs727088	European, European-Amerindian, Asian	NA^²^	[59, 60]

1q21–23	*FCGR2A*	ARG131HIS	European, European-Amerindian, African American	2.2	[23, 39, 40]

19p13.2	TYK2	rs280519 rs2304256 rs12720270	European	1.2	[40, 42]

3p21.3	*TREX1*	rs72556554	European, Asian, Hispanic, African	25	[61, 62]

Xq28	*MECP2-IRAK1*	rs2269368 rs17435 rs3027933 rs1734791	European, Chinese, Korean, European-Amerindian (Mexican)	1.4	[40, 63–65]

3p14.3	*PXK*	rs6445975 rs2176082	European	1.2	[23, 40]

2q24	*IFIH1*	rs1990760	European	NA^²^	[40]

11p15.5	*KIAA1542 (PHRF1)*	rs4963128	European	NA^²^	[23]

8p23.1	*XKR6*	rs6985109	European	NA^²^	[23]

6q21	*ATG5-PRMD1*	rs6568431, rs2245214 rs548234	European, Chinese	NA^²^	[23, 40, 66]

22q11.2	*UBE2L3*	s5754217	European, Chinese	1.2	[40, 45, 67]

5q33.3	*PTTG1*	rs2431099	European	1.2	[40, 45, 67]

6p21	*UHRF1BP1*	rs11755393	European	NA^²^	[40, 67]

5q32	*TNIP1*	rs7708392	European, Chinese, Thai, Japanese.	1.3	[38, 40, 67, 68]

7p15.2	*JAZF1*	rs849142	European	NA^²^	[40, 67]

7p21.3	*ICA1*	rs10156091	European	1.2	[23, 67]

1q24	*IL10*	rs3024505	European	NA^²^	[40, 67]

1q25.3	*NMNAT2*	rs2022013	European, Chinese	1.1	[23, 38]

11q23.3	*ETS1*	rs6590330	Chinese, Thai	NA^²^	[38, 46]

10q11.23	*WDFY4*	rs877819	Chinese, Thai	NA^²^	[38, 46]

7p12.2	*IKZF1*	rs4917014	Chinese	0.7	[38]

12q24.32	*SLC15A4*	rs10847697 rs1385374	Chinese	1.31	[38]

2p22.3	*RASGRP3*	rs13385731	Chinese	0.64	[38]

OR^1^: Approximate odds ratio.

NA^2^: Data not available.

**Table 2:** International Society of Nephrology/Renal Pathology Society (ISN/RPS) 2003 classification of LN [175].

*Class I minimal mesangial lupus nephritis*
Normal glomeruli by light microscopy, but mesangial immune deposits by immunofluorescence.

*Class II mesangial proliferative lupus nephritis*
Purely mesangial hypercellularity of any degree or mesangial matrix expansion by light microscopy, with mesangial immune deposits. May be a few isolated subepithelial or subendothelial deposits visible by immunofluorescence or electron microscopy, but not by light microscopy.

*Class III focal lupus nephritis*
Active or inactive focal, segmental, or global endo- or extracapillary glomerulonephritis involving <50% of all glomeruli, typically with focal subendothelial immune deposits, with or without mesangial alterations.

*Class IV diffuse lupus nephritis*
Active or inactive diffuse, segmental, or global endo- or extracapillary glomerulonephritis involving ≥50% of all glomeruli, typically with diffuse subendothelial immune deposits, with or without mesangial alterations. This class is divided into diffuse segmental (IV-S) lupus nephritis when ≥50% of the involved glomeruli have segmental lesions, and diffuse global (IV-G) lupus nephritis when ≥50% of the involved glomeruli have global lesions. Segmental is defined as a glomerular lesion that involves less than half of the glomerular tuft. This class includes cases with diffuse wire loop deposits but with little or no glomerular proliferation.

*Class V membranous lupus nephritis*
Global or segmental subepithelial immune deposits or their morphologic sequelae by light microscopy and by immunofluorescence or electron microscopy, with or without mesangial alterations. Class V lupus nephritis may occur in combination with class III or IV in which case both will be diagnosed Class V lupus nephritis show advanced sclerosis

*Class VI advanced sclerosis lupus nephritis*
≥90% of glomeruli globally sclerosed without residual activity.

## Abbreviations

SLE: Systemic lupus Erythematosus
LN: Lupus nephritis
ICs: Immune complexes
ENAS: Extractable nuclear antigen antibodies
IgG2: Immunoglobulin G subclass 2
PTEC: Proximal tubular epithelial cells
MPC-1: Monocyte chemoattractant protein-1
CAMs: Cell adhesion molecules
DCs: Dendritic Cells
Th2, th1, th17: Lymphocyte T helper 2, T helper1, and T helper 17
ESRD: End-stage renal disease
NZB: New Zealand Black mice
NZWF1: New Zealand White F1 mice
BXSB: In-bred strains of mice
MRL-Faslpr: Mice homozygous for the apoptosis-defective Faslpr mutation
ANAs: Antinuclear antibodies
FcR: Fc receptor of immunoglobulins
Fc*γ*R: Fc gamma receptor
BCR: B cell receptors
ICOS: Inducing costimulator
CTLA4: Cytotoxic T-Lymphocyte Antigen 4
(PD-1): Programmed death 1 costimulatory receptor
SHP-2: Nonreceptor protein tyrosine phosphatase
GWAS: Genome-wide association study
SNPs: Single nucleotide polymorphisms
HLA: Human leukocyte antigen complex
Anti dsDNA: Double-stranded anti-DNA antibodies
Anti RNP: Antiribonucleoprotein antibody
*Anti Ro/SS-A*,* Anti La/SS-B: *: Extractable nuclear antigens
GBM: Glomerular base membrane
IL-6: Interleukin-6
IL-1: Interleukin-1
TNF: Tumoral necrosis factor
PMNs: Polymorphonuclear cells
RANTES: Regulated upon activation, normal T-cell expressed, and secreted cytokine
TWEAK: TNF-related weak inducer of apoptosis
TGF: Transforming growth factor
PDGF: Platelet-derived growth factor
(APC): Antigen presenting cells
MAC: Complement membrane attack complex
FhT cells: Follicular helper T cells
TLR: Toll-like receptors
GM-CSF: Granulocyte macrophage colony stimulating factor
BlyS: B lymphocyte stimulator
CCR5: C-C chemokine receptor type 5
(NK): Natural Killer T cells
TCR: T cell receptors
NFAT: Nuclear factor of activated T cells
DN T cells: Double negative T cells
mABs: Monoclonal antibodies
BWF: 1hybrid between NZ and WNZ
TRAIL: TNF related apoptosis-inducing ligand
Tregs: T regulatory cells
RCTs: Randomized clinical trials.
